# TSPO–PET binding in vivo reflects increased phagocytic microglia at *post mortem* in people with frontotemporal dementia

**DOI:** 10.1007/s00401-026-03103-5

**Published:** 2026-10-22

**Authors:** Davi S. Vontobel, Kei Onn Lai, Mehtap Bacioglu, George Nolan, Daniel C. Maddison, Amelie Adamski, Julia Goddard, Noah L. Shapiro, Harry Crook, Tim Fryer, Young Hong, Sasvi Wijesinghe, Franklin Aigbirhio, Edward Avezov, Kieren S. J. Allinson, Annelies Quaegebeur, John T. O’Brien, James B. Rowe, Maria Grazia Spillantini, Maura Malpetti

**Affiliations:** 1 Department of Clinical Neurosciences, University of Cambridge, Cambridge, UK; 2 UK Dementia Research Institute, University of Cambridge, Cambridge, UK; 3 Department of Psychiatry, University of Cambridge, Cambridge, UK; 4 Cambridge University Hospitals NHS Trust, Cambridge, UK; 5 Department of Imaging and Pathology, University of Leuven, Louvain, Belgium

**Keywords:** TSPO–PET, Frontotemporal dementia, Microglia, CD68, Neuroinflammation, Post-mortem neuropathology

## Abstract

Brain inflammation is a key feature of frontotemporal dementia (FTD). Positron emission tomography (PET) of the 18 kDa translocator protein (TSPO) is widely used as an in vivo proxy for neuroinflammation, but whether the elevated signal reflects microglial, astrocytic or vascular pathology remains controversial, and this has not previously been resolved in FTD using matched *ante mortem* imaging and *post-mortem* tissue. We paired *ante mortem* [^11^C]PK11195 TSPO–PET with quantitative *post-mortem* neuropathology in 10 individuals with FTD (5 FTLD-tau, 5 FTLD–TDP) and 5 age- and sex-matched controls, combining CD68 immunohistochemistry across 17 brain regions, multiplex immunofluorescence pairing TSPO with microglial/macrophagic (IBA1, CD68), astrocytic (GFAP) and endothelial (CD31) markers in three frontotemporal cortical regions, and three-dimensional single-cell reconstruction of individual microglia. CD68 area fraction was elevated in FTD relative to controls, predominantly in white matter, and was positively associated with regional TSPO–PET-binding potential across the cohort (*β* = 8.66, *P* < 0.001), with comparable slopes in FTLD-tau and FTLD–TDP. Total tissue TSPO-area fraction was likewise increased in FTD and tracked regional PET binding in two independently processed slide sets. Of the four cell-type markers, only the CD68–TSPO co-localised fraction was positively associated with the PET signal (*β* = +0.0041, *P* = 0.015, *P*_FDR_ = 0.062), with null or negative associations for IBA1, GFAP and CD31. At single-cell resolution, IBA1+ microglia that were also CD68+ carried more absolute TSPO and were larger than CD68− microglia, without an increase in the TSPO fraction of cell volume. The elevated TSPO–PET signal in FTD, therefore, likely reflects an increased burden of CD68+ phagocytic microglia rather than TSPO expression in other cell types, supporting TSPO–PET as a microglial-burden biomarker across both FTLD-tau and FTLD–TDP.

**Supplementary Information:**

The online version contains supplementary material available at https://doi.org/10.1007/s00401-026-03103-5.

## Introduction

Frontotemporal dementia (FTD) encompasses a heterogeneous group of clinical syndromes, including behavioural variant FTD (bvFTD), semantic-variant primary progressive aphasia (svPPA), and non-fluent-variant primary progressive aphasia (nfvPPA) [12, 36]. Within this clinical heterogeneity, there is limited correspondence between phenotype and underlying neuropathological subtypes of frontotemporal lobar degeneration (FTLD) [13]. The most common pathological substrates are primary tauopathies (3R or 4R tau, FTLD-tau) and TAR–DNA-binding protein 43 (FTLD–TDP) [28], with rarer cases due to fused-in-sarcoma (FUS) proteinopathy.

Brain inflammation has emerged as a central feature of many neurodegenerative diseases, including FTD and other syndromes associated with FTLD [1, 5, 17]. PET ligands targeting the 18 kDa translocator protein (TSPO) are the most widely used in vivo PET markers to index brain inflammation in patients with these conditions [24]. A meta-analysis of 156 case–control studies confirmed elevated TSPO signal in core pathological regions across neurodegenerative conditions [7]. In sporadic and genetic FTD, there are disease-specific regional patterns of elevated TSPO–PET binding [22, 23]. The three clinical subtypes differ in their neuroanatomical TSPO–PET signal distribution [4], while increased signal in frontal regions predicts faster cognitive decline across all variants [23]. Elevated TSPO binding has also been observed across the broader FTLD spectrum [19], including the primary tauopathies progressive supranuclear palsy (PSP) and corticobasal degeneration [32, 34]. Increased TSPO binding in disease-specific regions predicts clinical decline, and correlates with tau pathology and its longitudinal progression [21, 22, 38].

However, the cellular basis of TSPO brain PET signal elevation in neurodegenerative diseases remains poorly resolved. Within the central nervous system, TSPO is primarily expressed by microglia, astrocytes, and endothelial cells [6, 14], with rare reports of neuronal expression [29]. An established paradigm held that microglial populations overexpress TSPO in neuroinflammatory states [9, 14]. Nutma et al. [30] challenged this view using human post-mortem tissue from multiple neurodegenerative and neuroinflammatory diseases, including Alzheimer’s disease (AD), amyotrophic lateral sclerosis, and multiple sclerosis, and found no evidence of increased per-cell TSPO expression in microglia or astroglia in disease when using single immunohistochemical markers (i.e., IBA1, CD68, HLA–DR). They concluded that the in vivo TSPO signal reflects changes in inflammatory cell density rather than per-cell upregulation. Work by Garland et al. [11] in AD demonstrated that TSPO-expressing microglia are predominantly CD68+, linking the signal to a phagocytic–microglial phenotype. In PSP, we recently reported a significant positive correlation between regional ^11^C-PK11195 binding and the increased burden of post-mortem CD68+ phagocytic microglia, and microglial TSPO levels, but not pathological astrocytic or endothelial TSPO [41].

The present study builds on this approach, to determine the cellular basis of TSPO–PET signal elevation in FTD, directly linking in vivo PET binding with *post-mortem* immunohistochemistry. In contrast to the clinically consistent PSP cohort with high clinicopathological correlation, we examine a clinically and pathologically heterogeneous FTD cohort with FTLD-tau and FTLD–TDP. We test the hypotheses that: (I) in vivo [^11^C]PK11195 PET binding is associated with *post-mortem* phagocytic microglia (specifically, CD68 +) burden regardless of the underlying FTLD pathology; (II) in vivo TSPO–PET signal reflects TSPO expression in phagocytic microglia over and above other cell types. The clinicopathological diversity of the cohort enables us to determine the pathological specificity of the TSPO–microglial relationship.

## Materials and methods

### Participants

Written informed consent was obtained from all participants. The protocols were approved by the National Research Ethics Service’s East of England Cambridge Central Committee, and the UK Administration of Radioactive Substances Advisory Committee (Neuropathology Research in Dementia protocol, Research Ethics Committee reference 16/WA/0240; Neuroimaging of Inflammation in Memory and Related Other Disorders (NIMROD), Research Ethics Committee reference 13/EE/0104).

Ten people with clinical diagnosis of FTD were included (Table 1); all underwent [^11^C]PK11195 PET scans during their lifetime and subsequently donated their brains to the Cambridge Brain Bank. Patients were clinically diagnosed with bvFTD (*n* = 3), nfvPPA (*n* = 4), or svPPA (*n* = 3) [12, 36]. Neuropathological assessments revealed the expected variability within the cohort: 5 cases of FTLD-tau (4R tau: corticobasal degeneration *n* = 2, PSP *n* = 1; 3R tau: Pick’s disease *n* = 1; FTLD-tau-other *n* = 1) and 5 cases of FTLD–TDP (TDP-43 Type A *n* = 1, Type B *n* = 1, Type C *n* = 3). Three of the ten patients were carriers of FTD-related gene mutations: patient 1 carried a mutation in the *MAPT* gene (c.915+16C>T, also coded as the *MAPT* 10+16 mutation); patient 3 carried a C9orf72 hexanucleotide repeat expansion (> 30 repeats), with the characteristic p62-immunoreactive cerebellar inclusions; patient 6 carried a c.388_391delCAGT, p.(Gln130Ser)fs *GRN* mutation.

**Table 1:** Demographic, clinical and neuropathological information for patients and controls

ID	Group	Sex	PET–PM (months)	Clinical syndrome	Post-mortem findings
Patient 1	FTD	F	91	bvFTD	FTLD-tau (MAPT 10+16 mutation carrier)
Patient 2	FTD	M	124	nfvPPA	FTLD-tau (CBD)
Patient 3	FTD	M	50	bvFTD	FTLD–TDP Type B (C9orf72 mutation carrier)
Patient 4	FTD	M	56	bvFTD	FTLD-tau (Pick’s disease)
Patient 5	FTD	F	100	svPPA	FTLD–TDP Type C
Patient 6	FTD	M	63	nfvPPA	FTLD–TDP Type A (GRN mutation carrier)
Patient 7	FTD	M	54	svPPA	FTLD–TDP Type C
Patient 8	FTD	M	86	svPPA	FTLD–TDP Type C
Patient 9	FTD	M	36	nfvPPA	FTLD-tau (PSP, Kovacs stage 6)
Patient 10	FTD	F	7	nfvPPA	FTLD-tau (CBD)
Control 1	Control	M	-	-	Normal brain (Braak I, CERAD 0, Thal phase 0)
Control 2	Control	F	-	-	No significant neurodegenerative disease (Braak I; mild age-related amyloid angiopathy)
Control 3	Control	M	-	-	No significant neuropathological changes
Control 4	Control	F	-	-	No neurodegenerative disease (Braak II)
Control 5	Control	M	-	-	No significant neurodegenerative pathology; Braak III tau, mild arterial sclerosis

Patients with FTD listed first (*n* = 10, ordered by ascending age at scan), age- and sex-matched controls below (*n* = 5, ordered by ascending age at death). PET–PM = interval between TSPO–PET scan and brain donation, in months

Brain tissues from five age- and sex-matched controls (Table 1) were included for group comparisons with patients at post-mortem. Controls were defined by the absence of significant neurodegenerative pathology at autopsy and Braak tangle stages ≤ III.

### Neuroimaging protocol and processing

Participants with FTD underwent in vivo TSPO–PET scans with [^11^C]PK11195 tracer, and structural MRI. MRI used Siemens Magnetom Tim Trio and Verio scanners (Siemens Healthineers, Erlangen, Germany) with an MPRAGE T1-weighted sequence, while PET used GE Advance and GE Discovery 690 PET/CT (GE Healthcare, Waukesha, USA) scanners. MRI and PET data were acquired and processed using previously described methods [3, 23]. Briefly, patients underwent a 3 T MRI scan, followed by a dynamic [^11^C]PK11195 PET scan for 75 min. Individual PET images were rigidly co-registered to the T1 MRI scans. The tissue time–activity curve was determined by supervised cluster analysis. Non-displaceable-binding potential (BPND) was calculated in cortical (Brodmann areas) and subcortical (modified n30r83 Hammersmith atlas, including brainstem parcellation and the cerebellar dentate nucleus) regions using a simplified tissue reference model with correction for vascular binding [21, 43]. Regional BPND values were extracted bilaterally: cortical values using the MRIcron digital Brodmann atlas, in which each Brodmann-area label encompassed both hemispheres, and non-Brodmann (subcortical and cerebellar) values as the mean of the left- and right-hemisphere parcels of the Hammersmith atlas, with brainstem parcels being unlateralised midline structures. No atrophy-based or partial-volume correction was applied to regional BPND estimates, to retain PET measures on an uncorrected tissue-volume basis comparable with the tissue-level histological area-fraction measures used for PET–post-mortem comparisons.

### Brain tissue sampling and preparation

The death-to-formalin post-mortem interval was 64.1 ± 12.4 h in individuals with FTD (median 64, range 39–83) and 57.4 ± 22.3 h in controls (median 51, range 32–92); the groups did not differ (Wilcoxon *W* = 33.5, *P* = 0.327). Tissue was formalin-fixed paraffin-embedded (FFPE) and stored at the Cambridge Brain Bank. FFPE blocks were sampled in 15 μm tissue sections from: Brodmann areas BA1/2/3 (jointly), BA4, BA6, BA7, BA17/18, BA21/22, BA46, amygdala, cingulate, cerebellar cortex, globus pallidus, putamen, hippocampal cortex, thalamus, medulla, midbrain, and pons. Cerebral tissue blocks were sampled from the left hemisphere, whereas cerebellar blocks were available from the left hemisphere in four patients and the right hemisphere in six patients.

### Immunohistochemistry protocol across all brain regions

Across all selected brain regions of patients and controls, DAB-based immunohistochemistry was performed to quantify CD68+ microglial load, phosphorylated tau (AT8), and TDP-43 aggregate burden (Online Resource 1, Table S5) with the same protocol as Wijesinghe et al. [41]. Whole-slide images were acquired on an Aperio AT2 whole-slide scanner (Leica) at ×40 magnification. Full-resolution images were processed in Halo (Indica Labs), where a tissue classifier (DenseNet V2, Indica Labs) was trained with manual annotations to differentiate between grey and white matter while simultaneously excluding non-tissue area, staining artefacts, and meningeal tissue. Grey- and white-matter areas were then colourimetrically quantified for CD68+ stain area.

### Immunofluorescence protocol in frontal, temporal and occipital regions

Fluorescent quadruple staining was performed for marker-co-localisation analyses in three frontotemporal cortical regions across all participants: inferior frontal gyrus (BA46) and temporal cortex (BA21/22), as primary affected in FTLD, and motor/premotor cortex (BA6) as a secondary affected region.

In brief, for co-staining fluorescent analysis, the FFPE slices were baked at 60 °C for 120 min. Following this, slides were rehydrated and deparaffinized with a series of baths; first washed in Xylene overnight, then moved to a separate tank with Xylene for 10 min. Slices were then washed sequentially in 100% Ethanol, 96% Ethanol, 70% Ethanol, 50% Ethanol, and finally RO H2O for 10 min each. Phosphate-buffered saline (PBS) solution is prepared from tablets (Thermo Fisher, 18912014) in reverse osmosis (RO) H2O. PBS-T is prepared by adding 0.05% Tween® 20 (Sigma-Aldrich) to PBS prepared as described above. The slides then proceeded to antigen retrieval in TRIS–EDTA (pH 9.0) at 90 °C for 20 min. After cooling to room temperature, slides were washed in PBS 3 times for 5 min per wash. Slides were laid flat outside of the solution and contoured with an Advanced hydrophobic PAP pen (Z672548, Sigma-Aldrich), then blocked with 2.5% Bovine Serum Albumin (BSA) in PBS-T for 60 min, after which primary antibody incubation was performed overnight with 2.5% BSA in PBS-T. On the following day, slides were washed in PBS 3 times for 5 min per wash. After which, TrueBlack^®^ Lipofuscin Autofluorescence Quencher (Biotium, 23,007) in 70% ethanol was applied to the slides for auto-fluorescent quenching for 30 s for the IBA1 stains and 45 s for the GFAP and CD31 stains. Subsequently, the slides were washed in PBS for 5 min 2 times. Slides were incubated with the appropriate secondary antibodies (see Online Resource 1, Table S6) in 2.5% BSA in PBS for 120 min. Following this incubation, slides were washed in PBS 3 times for 5 min each. Nucleic acids were stained with DAPI –4’,6-diamidino-2-phenylindole, dihydrochloride– (ThermoFisher, 62247) immediately after for 10 min. Finally, slides were washed in PBS 3 times for 5 min per wash and then coverslipped (Corning^®^, 2980–245) with Fluoromount-G™ Mounting Medium (Invitrogen, 00-4958-02).

The protocol used two vicinal slide sets per subject and region, prepared from sequential sections of the same FFPE block: **Set 1** (DAPI/IBA1/CD68/TSPO) and **Set 2** (DAPI/GFAP/CD31/TSPO). The TSPO antibody, dilution, and image-acquisition channel were the same across both slide sets. TrueBlack^®^ Lipofuscin Autofluorescence Quencher (Biotium) was used to minimise tissue autofluorescence. Primary and secondary antibodies, with dilutions and antigen retrieval buffers, are listed in Online Resource 1, Tables S5 and S6, respectively.

Fluorescent image acquisition for the co-localisation analysis was performed on a Zeiss Axioscan Z1 Slidescanner at 40×. For the single-cell co-localisation experiment, a Leica Stellaris 8 confocal microscope (40× water-immersion objective (HC PL APO 40x/1.10 W); FOV 228.16 × 228.16 μm) was used in Lightning™ deconvolution mode, with system-optimised Z-stacks acquired per image (depth ~10 μm; step ~0.36 μm). For quantification, six sub-images were extracted per slice and processed in ImageJ (Fiji) using custom BIOP JaCoP macros: background subtraction (rolling-ball radius = 13 μm, sliding paraboloid, no smoothing), Gaussian blur (*σ* = 2 μm, scaled units), and contrast enhancement (0.35% saturation) per channel. Co-localisation analyses used the BIOP JaCoP plug-in with Triangle thresholds applied to cell-marker channels (IBA1, CD68, GFAP) and Otsu thresholds applied to the TSPO channel. For the CD31–TSPO dataset, the four-channel images were first split to allow specific preprocessing of channel 4 (CD31): channels 1–3 were processed as above, while channel 4 underwent additional filtering (rolling-ball background subtraction radius = 120 μm, median filter radius = 2 μm, Gaussian blur *σ* = 2 μm, and local-maxima enhancement radius = 13 μm). The channels were then re-merged prior to co-localisation analysis, computed between CD31 and TSPO using Moments and Otsu thresholds, respectively.

### Three-dimensional single-cell quantification

Three-dimensional confocal image stacks were processed in Imaris 11 (Andor Oxford Instruments) to quantify intracellular TSPO and CD68 content at the level of individual IBA1+ microglia and CD68+ cells. Prior to object detection, each channel was preprocessed independently: a Gaussian filter (*σ* = 0.223 μm) was applied to the IBA1 channel; 3D median filters (kernel 3×3×1 voxels) were applied to the TSPO and CD68 channels, with an additional background subtraction step applied to the CD68 channel (*σ* = 57.088 μm).

For the IBA1-based pipeline, microglial cell bodies were segmented as 3D surfaces using a classifier trained on all four imaging channels, with IBA1 fluorescence as the primary reference. Surface grain size was set to 0.446 μm and the diameter of the largest sphere to 1.67 μm. Cell bodies were retained based on minimum voxel volume (≥ 6,333 voxels) and nucleus sphericity (≥ 0.46). TSPO+ and CD68+ puncta were detected as vesicle objects within each segmented cell body using local-contrast thresholding (estimated diameters: TSPO 1.0 μm isotropic; CD68 2.23 μm). The intracellular TSPO fraction and intracellular CD68 fraction were defined as the volume of vesicle-type objects occupying the cell-marker-positive cell volume, expressed as a proportion of total cell-body volume.

For the CD68-based pipeline, cell bodies were segmented from the CD68 channel alone using a classifier trained on this channel exclusively, with equivalent surface parameters. TSPO+ vesicle objects were detected within the resulting CD68+ cell volumes using an estimated diameter of 1.0 μm and local-contrast thresholding.

### Statistical analysis

Analyses were performed in R 4.5.3. Group descriptives used Wilcoxon rank-sum and Fisher's exact tests. Inferential models were frequentist linear mixed-effects models (LMEMs) with Kenward–Roger degrees of freedom; marginal means, slope contrasts, and pairwise comparisons used emmeans/emtrends. Single-cell Imaris tests used Wilcoxon rank-sum (exact) with Hodges–Lehmann shifts and 95% confidence intervals. Uncorrected *p* values are reported throughout; Benjamini–Hochberg FDR correction was applied where indicated. Full specifications of every model reported in the Results are provided in Online Resource 1, Table S7.

Random-effects structure: For LMEMs with a continuous within-subject predictor (TSPO–PET–BPND), the random-effects structure was selected per fit following Matuschek et al. [26]: three candidates were fit by REML and compared; random intercept only (1|id), random slope only (0+BPND|id), and correlated random intercept plus slope (1+BPND|id). Fits flagged as singular by lme4's isSingular were discarded; the lowest-AIC non-singular structure was retained. For binary group-only contrasts with no continuous within-subject predictor, the structure was fixed a priori at a subject random intercept. Packages used: tidyverse 2.0.0 (ggplot2 4.0.2, dplyr 1.2.1, tidyr 1.3.2, readr 2.2.0, purrr 1.2.1, tibble 3.3.1, stringr 1.6.0, forcats 1.0.1), readxl 1.4.5, janitor 2.2.1, lme4 2.0.1, lmerTest 3.2.1, emmeans 2.0.2, performance 0.16.0, ragg 1.5.2, patchwork 1.3.2, scales 1.4.0, Matrix 1.7.4, numDeriv 2016.8.1.1, estimability 1.5.1, mvtnorm 1.3.6, systemfonts 1.3.2, and textshaping 1.0.5.

Regional CD68 burden. CD68 ~ Group × Region + (1|id), with the Group main effect giving the global FTD-Control contrast and Group×Region testing regional heterogeneity; per-region contrasts via emmeans. A parallel model added Segmentation as a further interaction to localise effects to grey matter, white matter, or full tissue. BA4 was excluded from the FTD-vs-control comparison owing to absent control tissue coverage for this region.

TSPO–PET ~ CD68 burden and tissue TSPO area. CD68 ~ BPND + PET-to-post-mortem interval + (random effects), with a pathology–subgroup interaction (FTLD-tau vs FTLD–TDP) tested separately. Tissue TSPO area was first modelled per slide set (TSPO ~ Group + Region + (1|id)), then jointly across slide sets to compare slopes (TSPO ~ BPND + Slide Set + PET-to-post-mortem interval + (random effects)). Exploratory protein–aggregate associations (tau × CD68 in FTLD-tau; pTDP-43 × CD68 in FTLD–TDP) mirrored the BPND–CD68 structure.

Cell-type TSPO co-localisation. Per-marker LMEMs across BA6, BA46, BA21/22: Coloc ~ Group + Region + (1|id) for the group contrast, and Coloc ~ BPND + PET-to-post-mortem interval + (random effects) for the regional gradient, one per marker.

Single-cell Imaris metrics. Per-subject means of per-cell values, mean-of-ratios, GM and WM pooled, in Control (*n* = 4) and FTD (*n* = 10). Within-group: for each metric, per-subject CD68− vs CD68+ means were compared by exact paired Wilcoxon signed-rank test, separately in Control and FTD (Fig. 5). Between-group (supplementary): per-subject metrics were compared FTD vs Control by exact Wilcoxon rank-sum test with Hodges–Lehmann shifts and 95% CIs (Online Resource 1, Fig. S5). *p* values were BH–FDR corrected within each family.

## Results

### Cohort characteristics

Demographic, clinical and neuropathological details for the 15 participants in this study are summarised in Table 1. Ten patients with a clinical diagnosis of FTD (3 females, 7 males; mean age at death 71.1 years, with bvFTD, svPPA and nfvPPA phenotypes) and five age- and sex-matched controls (2 females, 3 males; mean age at death 73.6 years) were included for post-mortem group comparisons. The two groups did not differ in age at death (Wilcoxon rank-sum test: *W* = 22, *P* = 0.713; Hodges–Lehmann shift FTD-Control = − 2.0 years [95% CI − 13.0, +10.0]) or sex distribution (Fisher’s exact test: *P* = 1.00).

Neuropathological assessment confirmed substantial within-cohort variability: five patients had FTLD-tau pathology and five had FTLD–TDP pathology. Three of the ten patients were carriers of FTLD-related gene mutations: Patient 1 carried a *MAPT* mutation (c.915+16C>T, also coded as *MAPT* 10+16); Patient 3 carried a *C9orf72 hexanucleotide repeat expansion (*> *30 repeats, detected by repeat-primed PCR)*; and Patient 6 carried a c.388_391delCAGT, p.(Gln130Ser)fs *GRN* mutation. Controls were defined by the absence of significant neurodegenerative pathology at autopsy and Braak tangle stages ≤ III. Participant information provided in Table 1.

### Phagocytic microglia burden (CD68 +) is elevated in FTD

**Fig. 1:**
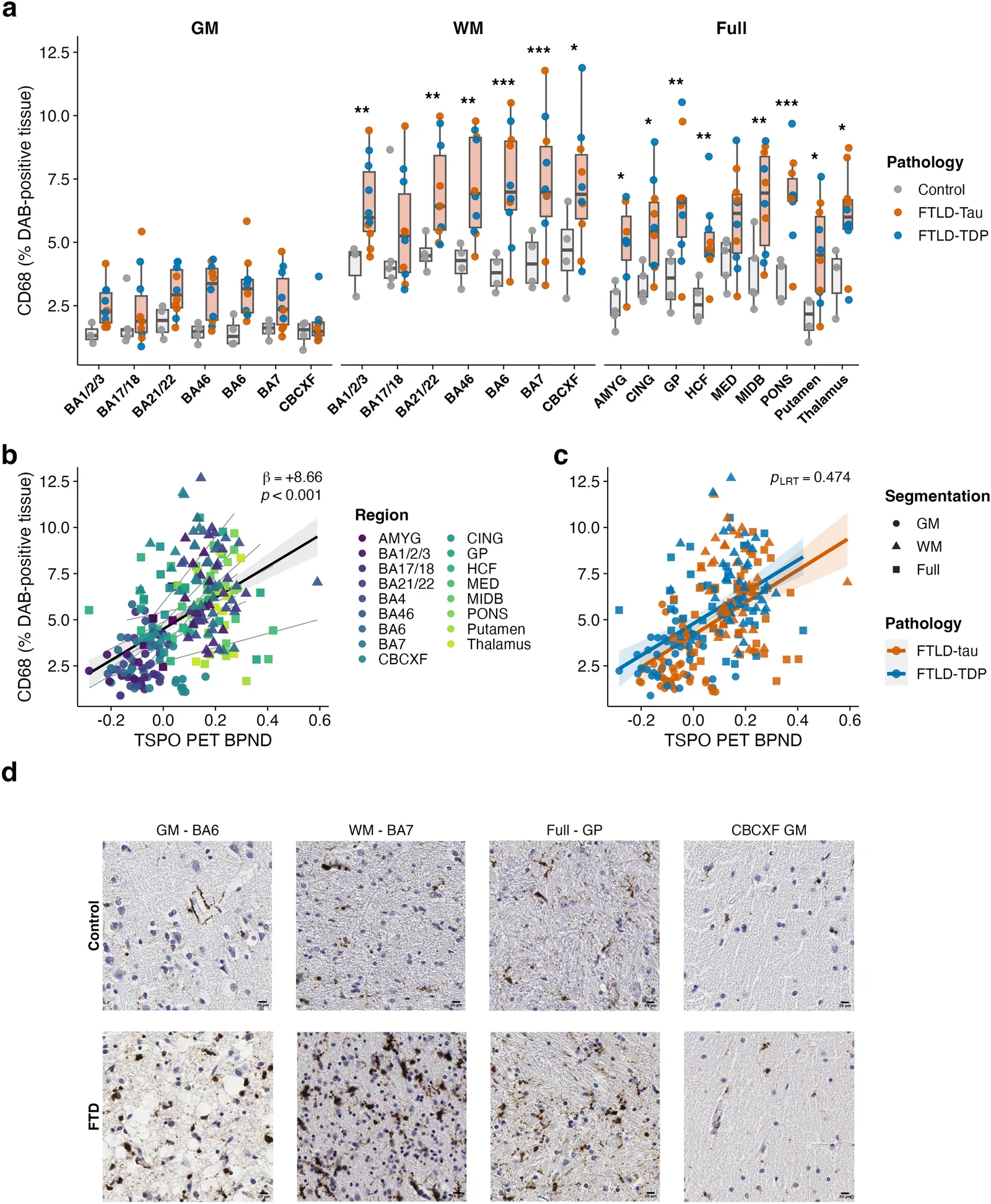
Post-mortem CD68 burden by anatomical region in patients with FTD vs controls, and its coupling with regional in vivo TSPO–PET binding. **a** CD68%DAB tissue area in patients with FTD vs Controls, (grey-matter and white-matter facets: BA1/2/3, BA17/18, BA21/22, BA46, BA6, BA7, CBCXF = cerebellum; Full = non-segmented facet: AMYG = amygdala, CING = cingulate, G*P* = globus pallidus, HCF = hippocampal cortex, MED = medulla, MIDB = midbrain, Pons, Putamen, Thalamus). Asterisks mark FTD-vs-Control contrasts at *P* <0.05 *, *P* <0.01 **, *P* <0.001 ***. BA4 was excluded from the Control x FTD comparison due to absent control coverage; CD68 ~ Group × Region + (1|id), Type III *F* tests with Kenward–Roger df: group main effect F(1, 13.9) = 14.63, *P* = 0.002, *β*_(FTD−Ctrl) = +2.21, marginal R^2^ = 0.22; Group × Region F(15, 279.1) = 0.47, *P* = 0.956; *N* = 323 observations/15 subjects. **b** Regional CD68 area fraction vs regional TSPO–PET binding (BPND) in all 10 patients with FTD; per-subject linear fits (grey) overlaid with the overall LMEM trend (black, 95% CI ribbon); CD68 ~ BPND + PET_PM_Interval + (1|id): *β*_BPND = +8.66, SE = 0.86, *t* = 10.11, *P* < 0.001; marginal R^2^ = 0.30, conditional R^2^ = 0.45; *N* = 247 observations/10 subjects. **c** Same LMEM as panel b with pathology-specific fits overlaid; CD68 ~ PathClass × BPND + PET_PM_Interval + (1|id): slope-equality LRT χ^2^ = 0.514, *df* = 1, *P* = 0.474; FTLD-tau *β* = 9.36, FTLD–TDP *β* = 8.05. **d** Representative CD68 DAB-immunohistochemistry images, one FTD case and one matched control per region; scale bar 20 μm. From left to right: grey-matter BA6, white-matter BA7, globus pallidus, and cerebellar grey matter, scale bar = 20 μm

We tested whether CD68 burden at *post mortem* differed between patients with FTD and controls using a linear mixed-effects model (LMEM) with group (FTD vs controls), region, and their interaction as fixed effects, and a random intercept for subject. Type III *F* tests with Kenward–Roger degrees of freedom were applied across 16 cortical, subcortical, brainstem and cerebellar regions (BA4 excluded due to lack of control coverage). CD68 area fraction was elevated in patients with FTD compared to controls (group main effect: *F*(1, 13.9) = 14.63, *P* = 0.002; *β*_(FTD-Ctrl) = +2.21, marginal *R*^2^ = 0.22; Fig. 1).

Post hoc analyses with a segmentation-aware companion model identified regions, where CD68 area fraction was higher in patients than controls (emmeans package; see Table 2 for per-region contrasts). Across cortical Brodmann regions, elevation in CD68 area fraction in patients was predominantly in white matter: CD68 differences in white matter were significant in BA6, BA7, BA21/22, BA46, BA1/2/3 and the cerebellum (CBCXF), while in contrast, no cortical grey-matter region reaching significance (*P* > 0.05). Concordantly, CD68 area fraction in cerebellum was higher in patients than controls in white-matter regions (*P* = 0.011) but not in grey matter (*P* = 0.808). Primary visual cortex (BA17/18) did not show a consistent group difference in grey-matter or white-matter areas. When analysing the full-quantified regions (no segmentation between white and grey matter), we found increased CD68 burden in the anterior cingulate, globus pallidus, hippocampal cortex, putamen, thalamus, amygdala, midbrain and pons, with medulla at trend (*P* = 0.065). However, when each compartment was modelled separately across the segmented cortical regions, the group elevation was significant in both grey matter (*F*(1, 13.5) = 8.48, *P* = 0.012; *β* = +1.07, 95% CI [0.28, 1.87]) and in white matter (*F*(1, 13.5) = 11.38, *P* = 0.005; *β* = +2.50, 95% CI [0.91, 4.10]), with no group × region interaction in either compartment, indicating that CD68 burden is elevated in both compartments yet the larger absolute differences lie in white matter (Online Resource 1, section A7).

**Table 2:** Group comparisons on CD68 area-fraction values: results from linear mixed-effects model (Region × Segmentation x Groups)

Region	Tissue type	*β* (FTD-Ctrl)	P value
BA6	GM	+1.54	0.097
BA6	WM	+3.30	0.0006*
BA7	GM	+1.26	0.172
BA7	WM	+3.23	0.0007*
BA17/18	GM	+0.55	0.530
BA17/18	WM	+0.76	0.389
BA21/22	GM	+1.36	0.143
BA21/22	WM	+2.57	0.006*
BA46	GM	+1.51	0.103
BA46	WM	+3.01	0.002*
BA1/2/3	GM	+1.28	0.199
BA1/2/3	WM	+2.69	0.008*
CBCXF	GM	+0.22	0.808
CBCXF	WM	+2.39	0.011*
PONS	Full	+3.20	0.0005*
MIDB	Full	+2.93	0.001*
MED	Full	+1.63	0.065
GP	Full	+2.92	0.002*
Putamen	Full	+2.42	0.010*
Thalamus	Full	+2.51	0.013*
HCF	Full	+2.50	0.008*
AMYG	Full	+2.19	0.018*
CING	Full	+2.25	0.012*

Significance grades reflect linear mixed-effects model posthoc emmeans-contrasts: * *P* < 0.05. Tissue type: *GM* grey matter, *WM* white matter, *Full* non-segmented sampling

### In vivo TSPO–PET binding correlates with *post-mortem* phagocytic–microglial markers

To determine whether TSPO-PET-binding potential (BPND) reflects phagocytic–microglial/macrophage burden (CD68+ at post-mortem), an LMEM modelled CD68 area fraction as the outcome with regional BPND and PET-to-post-mortem interval as fixed-effect predictors and a subject-level random intercept. Across all 10 patients with FTD, the TSPO–PET signals were positively associated with CD68 area fraction (*β* = 8.66, SE = 0.86, *t* = 10.11, *P* < 0.001; marginal R^2^ = 0.30, conditional R^2^ = 0.45; Fig. 1b). The PET-to-post-mortem interval was not a significant predictor.

To test whether the *ante mortem* TSPO-signal–CD68 association differed by pathology, we extended the model with an interaction between regional BPND and pathology subgroup*.* The interaction did not improve fit (LRT χ^2^ = 0.514, *df* = 1, *P* = 0.474), and per-pathology slopes were comparable for FTLD-tau and FTLD–TDP cases (FTLD-tau *β* = 9.36, 95% CI [6.94, 11.79]; FTLD–TDP *β* = 8.05, 95% CI [5.69, 10.42]; slope contrast TDP-Tau = − 1.31, *P* = 0.445; Fig. 1c). The TSPO–PET–CD68 coupling is, therefore, observed across both proteinopathies with comparable magnitude. As a follow-up analysis we reused neuropathological diagnosis slides (AT8 pTau and pTDP-43 stains) from the cohort for verification with the TSPO–PET signal and CD68 staining. TSPO–PET did not meaningfully correlate with protein–aggregate load in either FTLD subgroup; however, AT8+ tau and CD68 displayed a mild positive association pre FDR correction (Online Resource 1, Fig. S1a).

### Tissue TSPO area is elevated in FTD and correlates with regional TSPO–PET binding

Given the positive association between in vivo* TSPO–PET* and *post mortem* CD68, we tested whether total tissue TSPO area at *post mortem* was elevated in people with FTD relative to controls and associated with regional TSPO–PET in vivo signal. Two vicinal slide sets per subject (Set 1: IBA1/CD68/TSPO; Set 2: GFAP/CD31/TSPO) were quantified across BA6, BA21/22 and BA46, with TSPO area expressed as ratio of TSPO+ area over total tissue area (TSPO-area fractio*n* = TSPO area/Total area) and analysed independently per slide set.

**Fig. 2:**
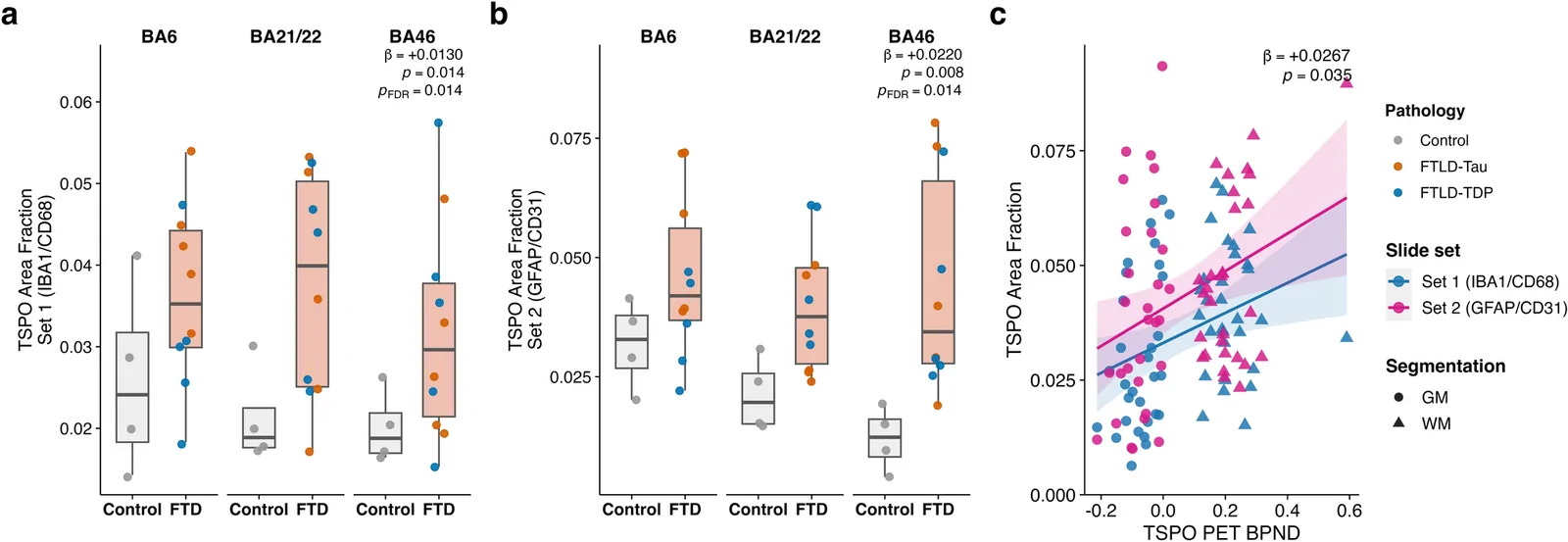
Post-mortem tissue TSPO-area fraction is increased in frontotemporal regions and tracks regional in vivo TSPO–PET binding in FTD. **a** FTD-vs-Control contrast for Set 1 (IBA1/CD68 stain); box fill salmo*n* = FTD, light grey = Control; points coloured by pathology subgroup; Set 1 (IBA1/CD68): *β* = +0.0130, *P* = 0.014, *P*_FDR = 0.014. **b** FTD-vs-Control contrast for Set 2 (GFAP/CD31 stain); same encoding and model as panel a; Set 2 (GFAP/CD31): *β* = +0.0220, *P* = 0.008, *P*_FDR = 0.014. **c** Joint paired-subject TSPO-area × TSPO–PET scatter across all 10 patients with FTD, both slide sets overlaid on the same axes; ribbon colour = slide set (Set 1 IBA1/CD68 = blue, Set 2 GFAP/CD31 = magenta); shape = segmentation (grey matter = circle, white matter = triangle); Norm_AreaB ~ BPND × SlideSet + PET_PM_Interval + (1|id); Set 1 *β* = +0.0267, *P* = 0.036, *P*_FDR = 0.036. Set 2 (GFAP/CD31) *β* = +0.0348, *P* = 0.007, *P*_FDR = 0.013

TSPO-area fraction was elevated in patients with FTD relative to controls in both slide sets (Set 1, IBA1/CD68: *β*_(FTD-Ctrl) = +0.013, *P* = 0.014, *P*_FDR = 0.014; Set 2, GFAP/CD31: *β* = +0.022, *P* = 0.008, *P*_FDR = 0.014; Fig. 2a, b). The two slide sets agreed in direction and magnitude despite being processed independently. The Region main effect was not significant in either slide set (Set 1, IBA1/CD68: *F*(2, 26.4) = 1.11, *P* = 0.344; Set 2, GFAP/CD31: *F*(2, 26.1) = 1.31, *P* = 0.287).

Within FTD, *post-mortem* regional TSPO-area fraction was positively associated with regional TSPO–PET signal in both slide sets; Set 1, IBA1/CD68: *β*_BPND = +0.0267, *P* = 0.036, *P*_FDR = 0.036; Set 2, GFAP/CD31: *β*_BPND = +0.0348, *P* = 0.007, *P*_FDR = 0.013; slope contrast Set 1–Set 2 = − 0.008, *P* = 0.642; Fig. 2c). PET-to-post-mortem interval was a covariate of no interest.

### TSPO expression co-localises with phagocytic microglia

We then tested whether co-localisation of immunofluorescence signals between TSPO and each of four cell-type-specific markers are elevated in the FTD group. These markers include IBA1 (microglia/macrophage membrane), CD68 (microglia/macrophages lysosomes), GFAP (astroglial cytoskeleton), and CD31 (vascular endothelial cells/blood vessels). Image-level co-localised area (TSPO and marker double-positive area/total tissue area; ratio of co-localised area over total area) was modelled per marker by a binary LMEM (FTD vs Control) with region as a fixed effect and a subject random intercept.

**Fig. 3:**
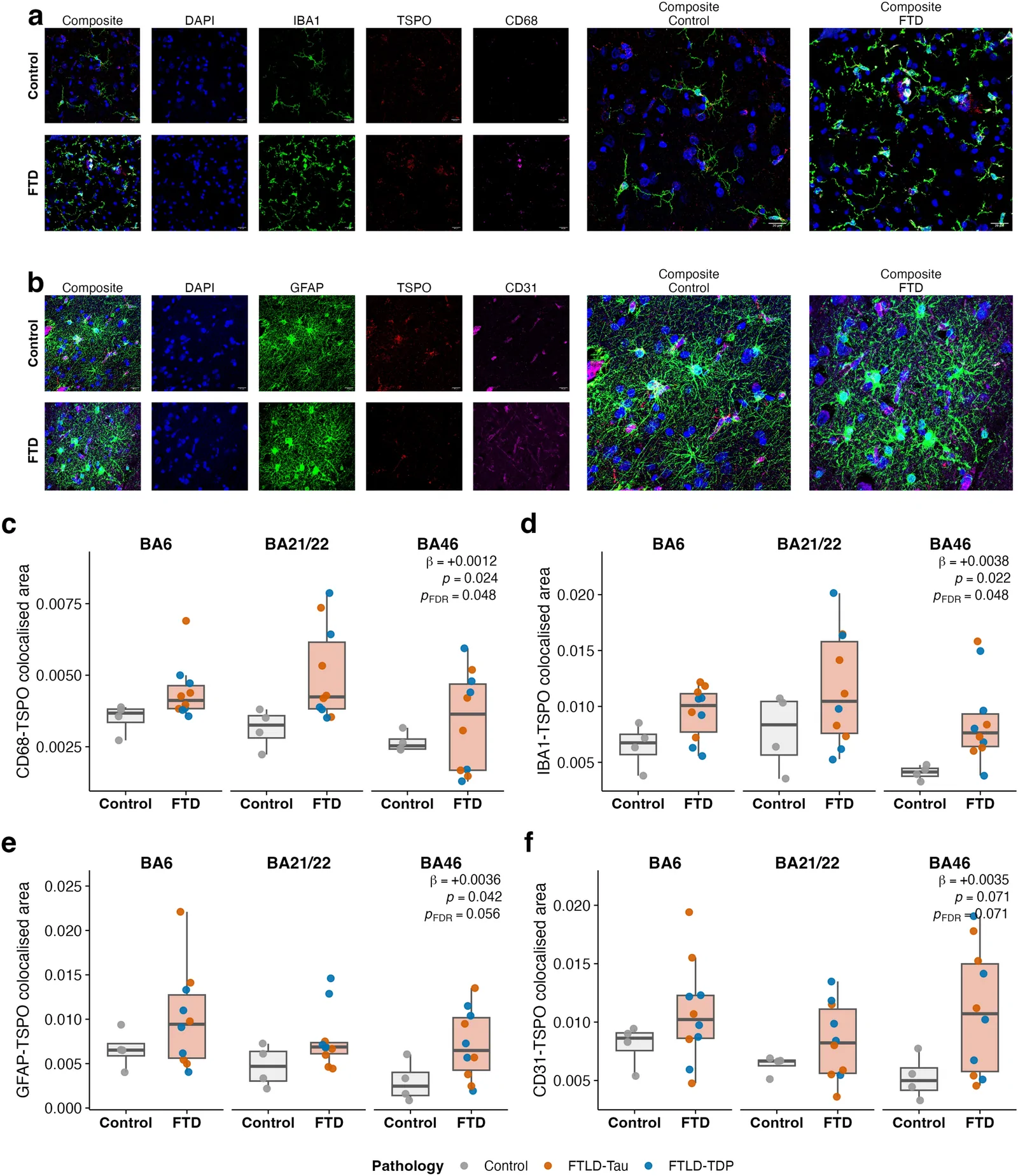
Marker–TSPO co-localised area is elevated in FTD in microglia. **a** Representative immunofluorescence in BA46 in FTD and matched controls across DAPI/IBA1/TSPO/CD68 single-channel columns and the merged composite; scale bar = 20 μm. **b** Representative immunofluorescence in BA46, same layout as panel a for DAPI/GFAP/TSPO/CD31; scale bar = 20 μm. **c–f** Coloc_by_Total ~ FTD_vs_Control + Region + (1|id); FTD−Control *β* per marker with BH–FDR across the 4-marker family. **c** CD68-TSPO co-localised area (Coloc/Total Area) compared between FTD and Controls in BA6, BA21/22 and BA46; box fill salmo*n* = FTD, light grey = Control; points coloured by pathology subgroup; CD68–TSPO: *β* = +0.0012, *P* = 0.024, *P*_FDR = 0.048. **d** IBA1–TSPO co-localised area; IBA1–TSPO: *β* = +0.0038, *P* = 0.022, *P*_FDR = 0.048. **e** GFAP–TSPO co-localised area; GFAP-TSPO: *β* = +0.0036, *P* = 0.042, *P*_FDR = 0.056. **f** CD31–TSPO co-localised area; CD31–TSPO: *β* = +0.0035, *P* = 0.071, *P*_FDR = 0.071

Area fraction of co-localisation with TSPO was elevated in patients with FTD relative to controls for three markers (CD68: *β*_(FTD-Ctrl) = +0.0012, *P* = 0.024, *P*_FDR = 0.048; IBA1: *β* = +0.0038, *P* = 0.022, *P*_FDR = 0.048; GFAP: *β* = +0.0036, *P* = 0.042, *P*_FDR = 0.056; Fig. 3c–f), while the endothelial CD31–TSPO co-localised area was not (CD31: *β* = +0.0035, *P* = 0.071, *P*_FDR = 0.071). However, with multiple-comparison correction across the 4-marker family, comparisons in TSPO co-localisation with CD68 and IBA1 markers remained significant (*P*_FDR = 0.048 each), while TSPO co-localisation areas with GFAP and CD31 markers were not statistically significant (*p*>0.05).

### TSPO–PET binding only correlated with phagocytic microglia burden

To identify which cellular sources of TSPO drive the in vivo PET signal in patients with FTD, we tested the association between co-localisation area for each markers and regional TSPO–PET signal, fitting an LMEM per marker across BA6, BA21/22 and BA46. The random-effects structure was selected by the Matuschek ladder per marker.

**Fig. 4:**
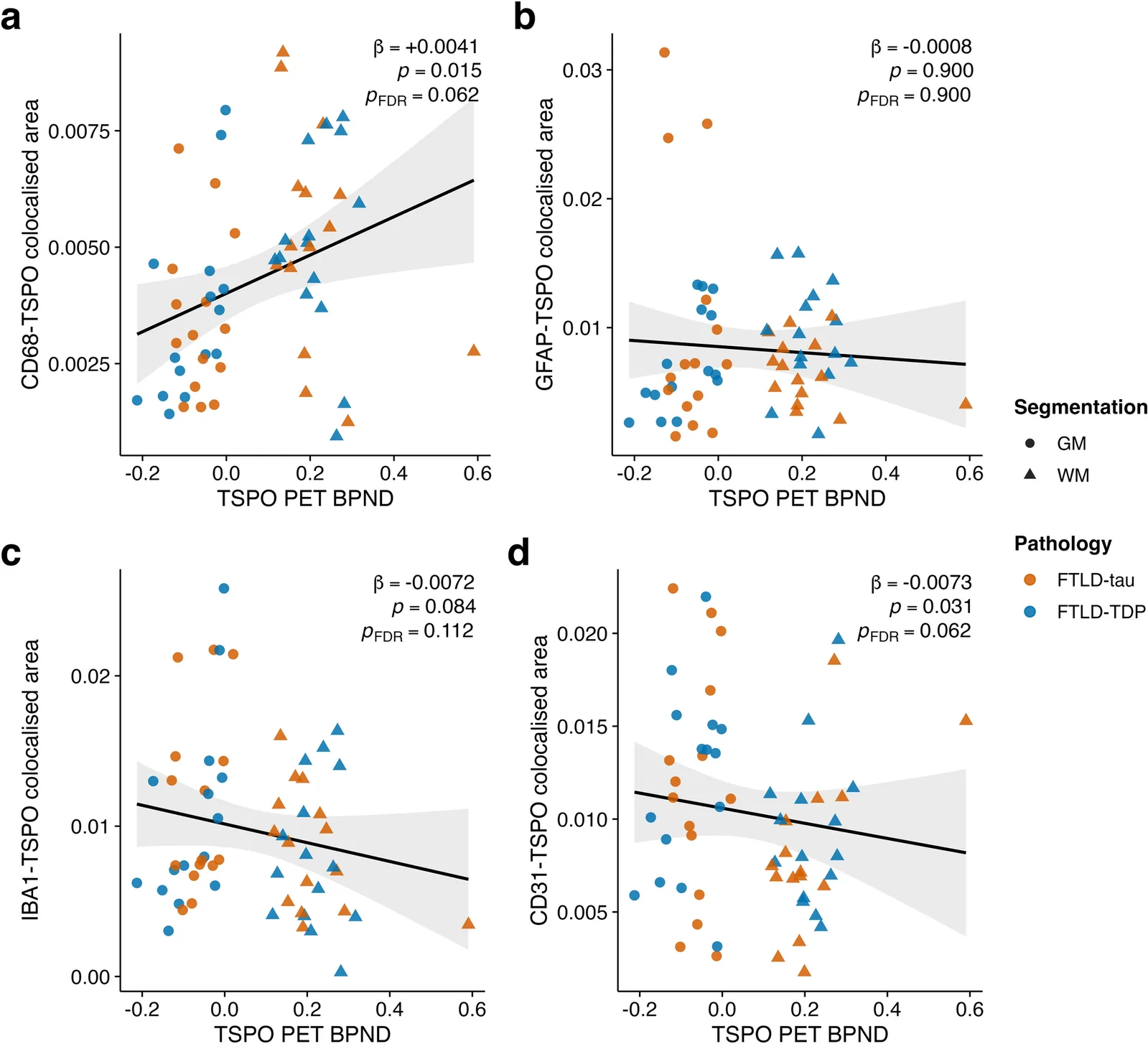
Cell-type-specific marker–TSPO co-localised area modelled against regional TSPO–PET in FTD**.** Coloc/Total Area vs regional BPND in all 10 patients with FTD, fitted per marker. Points coloured by pathology subgroup (FTLD-tau orange, FTLD–TDP blue); shape = segmentation (grey matter = circle, white matter = triangle, Full = square), (**a–d**) Coloc_by_Total ~ BPND + PET_PM_Interval + random effects, fitted per marker with Matuschek-selected structure (CD68, IBA1, CD31 = (1|id); GFA*P* = (1 + BPND|id)); BH–FDR across the 4-marker family. **a** CD68–TSPO co-localisation; *β*_BPND = +0.0041, *P* = 0.015, *P*_FDR = 0.062. **b** GFAP–TSPO co-localisation; *β*_BPND = −0.0008, *P* = 0.900, *P*_FDR = 0.900. **c** IBA1–TSPO co-localisation; *β*_BPND = −0.0072, *P* = 0.084, *P*_FDR = 0.112. **d** CD31–TSPO co-localisation; *β*_BPND = −0.0073, *P* = 0.031, *P*_FDR = 0.062

Across all patients, regional TSPO–PET signal was positively associated with phagocytic microglia (CD68–TSPO co-localised fraction: *β* = +0.0041, *P* = 0.015, *P*_FDR = 0.062) and negatively associated with the endothelial CD31–TSPO co-localised fraction (*β* = − 0.0073, *P* = 0.031, *P*_FDR = 0.062) before FDR correction, with a graphical-trend-level negative association for IBA1–TSPO (*β* = − 0.0072, *P* = 0.084, *P*_FDR = 0.112) and no association for GFAP–TSPO (*β* = − 0.0008, *P* = 0.90, *P*_FDR = 0.90; Fig. 4).

The four-marker pattern positions phagocytic microglia (CD68 +) as the cell-type marker whose co-localisation with TSPO tracks the in vivo PET signal positively, with null or negative associations for IBA1, GFAP, and CD31. Exploratory compartment-specific analyses indicated a differential association between PET signal and microglial TSPO co-localisation across compartments. A grey-matter/white-matter decomposition (Online Resource 1, Fig. S3) shows the CD68 positive coupling concentrated in grey matter (GM *β* = +0.013, *P* = 0.052; WM *β* = − 0.009, *P* = 0.027; TSPO–PET signal × Segmentation interaction LRT *P* = 0.007), with the same compartment-specific structure for IBA1 (GM *β* = +0.048, *P* = 0.006; WM *β* = − 0.016, *P* = 0.122; LRT *P* = 0.001). GFAP and CD31 show no significant Segmentation interaction (LRT *P* = 0.310 and 0.88, respectively).

### Single-cell metrics of microglial TSPO in FTD

**Fig. 5:**
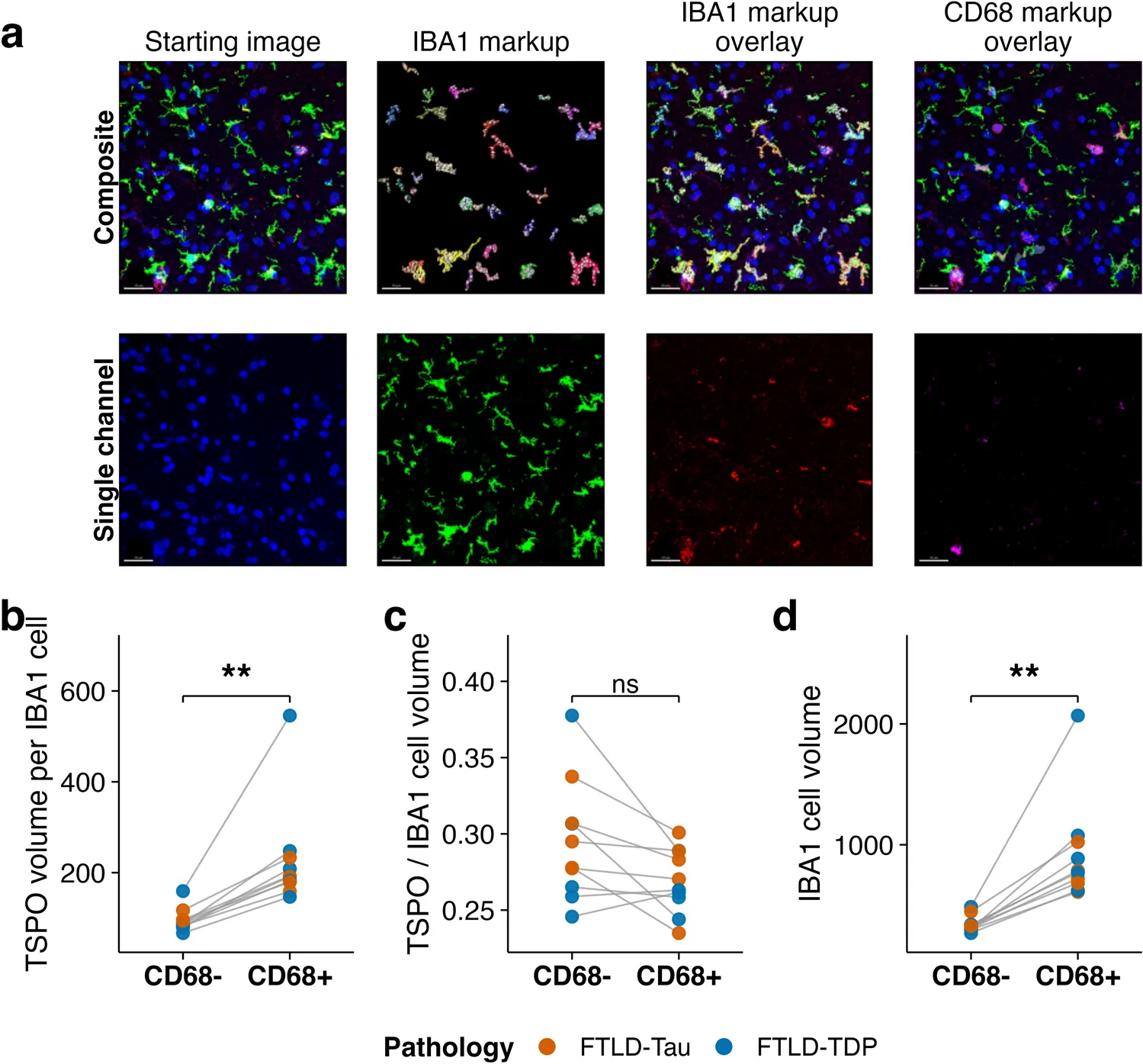
CD68+ microglia carry more TSPO in proportion to their size. Colour palette throughout: Control = grey, FTLD-tau = orange, FTLD–TD*P* = blue. **a** Representative Imaris three-dimensional single-cell segmentation from BA46 tissue from an FTD case: an IBA1+ cell-body surface with intracellular TSPO and CD68 vesicle objects rendered within the cell volume; scale bar = 30 μm. **b–d** Within IBA1+ microglia, each cell was classified CD68− or CD68+ (CD68 volume 0 vs > 0) and per-subject means compared between classes by paired Wilcoxon signed-rank test, analysing Control (**b**–**d**, *n* = 4) and FTD (e–g, *n* = 10) separately. (**b**) Mean TSPO volume per IBA1+ cell; (**c**) TSPO fraction of cell volume; (**d**) IBA1+ cell volume. Each grey line links one subject's CD68− and CD68+ means; points coloured by pathology subgroup. Brackets show BH–FDR–adjusted significance within each metric (** P_FDR < 0.01; ns, not significant). In FTD, CD68+ microglia carried more TSPO per cell (10/10 subjects; V = 55, *P* = 0.002, P_FDR = 0.004) and were larger (10/10; V = 55, *P* = 0.002, P_FDR = 0.004), the TSPO fraction of cell volume was not higher (8/10; V = 6, *P* = 0.027, P_FDR = 0.055, ns)

To quantify TSPO expression per microglial cell, we performed 3D rendering of IBA1+ cells in confocal images. Variables were aggregated to per-subject means across quantification samples. Total TSPO volume per cell was acquired. Within IBA1+ microglia we classified each cell as CD68− or CD68+ (CD68 volume 0 vs > 0) and compared, per subject, the mean TSPO per cell, the TSPO fraction of cell volume, and cell size, analysing Control and FTD separately (Fig. 5b–g).

In FTD, CD68+ microglia carried more absolute TSPO than CD68− microglia (10/10 subjects; *P* = 0.002, *P*_FDR = 0.004) and were larger (10/10; *P* = 0.002, *P*_FDR = 0.004), whereas the TSPO fraction of cell volume was not higher, if anything lower (8/10; *V* = 6, *P* = 0.027, *P*_FDR = 0.055, ns). Controls showed the same directional pattern for each metric, although none of the within-group comparisons reached significance in the small control subset (n=4, all *P*>0.05). IBA1+ cell counts did not differ between the selected confocal fields from the two groups (*P* = 0.288; Online Resource 1, Fig. S5), excluding a sampling or cell-density bias within the fields. Within the sampled fields, the proportion of IBA1+ microglia classified as CD68+ was numerically higher in FTD (median 0.45) than in controls (median 0.23; exact Wilcoxon *W* = 33, *P* = 0.076; Hodges–Lehmann shift +0.19, 95% CI [− 0.07, +0.38]; Online Resource 1, Fig. S5), but this exploratory comparison did not reach statistical significance. These field-level cell counts are presented to describe the composition of the single-cell imaging subset and were not used as estimates of regional microglial density.

## Discussion

The principal finding of this study is that in both FTLD-tau and FTLD–TDP forms of frontotemporal dementia, increased TSPO–PET binding is associated with CD68+ phagocytic microglia. *Post-mortem* TSPO signal was also tightly linked with the in vivo TSPO–PET signal, across patients and brain regions. Overall, there was an increase in microglial area (both IBA1+ and CD68 +) co-localised with TSPO, while astrocytic and endothelial TSPO remained comparable to controls (arguable trend in GFAP+ astrocytes). However, only the CD68–TSPO area was positively linked with the in vivo TSPO–PET signal (before FDR correction). Finally, within BA46 exclusively, we analysed the intracellular contents of the IBA1+ cells and found that CD68+ cells were both larger than their CD68- counterparts and expressed more absolute TSPO.

Elevated phagocytic–microglial protein burden at *post mortem* is a recurring neuropathological feature across neurodegenerative conditions. CD68-immunoreactive microglia are increased in Alzheimer’s disease [16], Parkinson’s disease [8], and all major subtypes of frontotemporal lobar degeneration [42]. Our finding of increased CD68 burden in both FTLD-tau and FTLD–TDP accords with this literature and extends it to a directly PET-matched FTD cohort at post-mortem. The positive association between in vivo TSPO–PET signal and CD68 area fraction at *post mortem* across the brain expands the previous PET-to-*post-mortem* evidence in PSP, where we reported strong positive relationship between TSPO–PET and CD68 burden [41]. In the present FTD cohort, the random-effects model selected by AIC similarly retained a subject-level intercept only [26]. The preserved directionality and comparable magnitude of the association between in vivo TSPO–PET–BPND and post-mortem TSPO expression across the two independent cohorts suggests that the relationship may generalise across all FTLD subtypes, including 4R-tau PSP and in our heterogeneous FTLD-tau + FTLD–TDP. Previous *post-mortem* analysis in AD demonstrated that the dominant TSPO sources within the temporal lobe were CD68+ microglia [11]. These studies in combination suggest that the TSPO–PET signal captures a pathology-independent microglial component across different neurodegenerative diseases.

The concordance between *ante mortem* TSPO–PET-binding and *post-mortem* TSPO-immunoreactive area-fraction positions TSPO as a marker of sustained neuroinflammatory burden rather than a transient signal that resolves with disease progression in FTD. Previous longitudinal TSPO–PET studies have demonstrated stable signals over 1-year intervals in multiple sclerosis white matter hyperintensities [31] or increasing signals at follow-up in AD in disease-associated areas [10, 20]. This contrasts with transient elevation following acute cerebrovascular injury [18], illustrating important, if a dash obvious, differences between chronic brain inflammation in neurodegeneration and the acute inflammatory responses to discrete insults.

The fluorescent co-localisation analyses showed that the TSPO–PET signal is preferentially associated with CD68+ microglia. Despite an observed increase in both CD68–TSPO and IBA1–TSPO areas within the FTD cohort, CD68–TSPO co-localisation was positively associated with regional TSPO–PET in vivo signal, while GFAP–TSPO and IBA1–TSPO co-localised area fractions were not. Although this CD68–TSPO association did not survive multiple-comparison correction (*P*_FDR = 0.062), it recapitulated the larger 17-region CD68 burden association with in vivo TSPO–PET signal. In this context, our findings suggest that the in vivo TSPO–PET signal in patients with FTD is driven by phagocytic microglia, rather than TSPO expression in other cell types. This pattern is concordant with previous post-mortem-only observations in AD, where CD68–TSPO co-localisation was higher than the TSPO co-localisation with IBA1, HLA–DR, MSR-A, and CD64+ microglia/macrophages in the temporal cortex [11]. The relatively weak negative association between TSPO–PET-binding and the CD31–TSPO co-localised area fraction may reflect age- and disease-related loss of endothelial TSPO-binding sites [37, 39]; however, further quantitative pathology studies are needed to comprehend this signal decoupling.

Our data also highlight a dissociation between grey and white matter in the disease-level CD68 burden and the PET-coupling signal. In the segmentation-aware group model (Fig. 1a), the CD68 elevation in the FTD cerebrum was white-matter dominant: CD68 was significantly elevated in the white matter underlying every segmented cortical region except primary visual cortex, as well as in the cerebellum, whereas no cortical grey-matter contrast reached significance. However, when each compartment was modelled separately, CD68 burden was elevated in grey matter as well as in white matter, with the larger absolute differences in white matter on its higher baseline (Online Resource 1, section A7). This pattern accords with the white-matter neuroinflammatory burden described in FTLD, where CD68 burden exceeds grey matter in all groups including controls and is elevated in patients in both compartments [42], with the higher baseline microglial density of white matter [27] and may in part reflect microglial responses to myelin and axonal degeneration downstream of cortical neurodegeneration.

Importantly, the FTD-control analysis and the PET-coupling analysis addressed different questions. CD68 burden was elevated in both grey and white matter when each compartment was modelled across regions, although the larger absolute group difference was observed in white matter. By contrast, the co-localisation analysis tested whether regional variation in marker-associated TSPO tracks regional PET binding within the FTD cohort, rather than whether either compartment shows the largest FTD-control difference. The association between TSPO–PET and co-localised marker area was compartment-dependent for both microglial markers: modelled separately, the coupling was positive in grey matter (but not in white matter. The TSPO–PET-by-compartment interaction was significant for both microglial markers (Online Resource 1, Fig. S3). In contrast, the corresponding compartment-specific analysis of total post-mortem TSPO-area fraction showed concordantly positive slopes in grey and white matter, and inclusion of a TSPO–PET-by-compartment interaction did not improve model fit (Online Resource 1, Fig. S2). Thus, in this dataset, the compartment-specific pattern was observed for marker–TSPO co-localisation rather than total tissue TSPO. The basis for this pattern remains uncertain. The immunofluorescence analysis was confined to three cortical regions and quantified micrometre-scale spatial co-localisation, whereas PET provides a regional signal at millimetre-scale resolution. In addition, immunofluorescence and DAB-based immunohistochemistry quantify related but non-identical features of tissue pathology. Finally, cerebral post-mortem tissue was sampled from one hemisphere only; in FTD, pathological burden and inflammatory changes may be lateralised. These factors may contribute to variability in the compartment-specific PET–histology associations but cannot be disentangled in the present study. Dedicated in vivo analyses that distinguish grey- and white-matter TSPO–PET signal will be important to clarify the compartment-specific determinants of TSPO–PET in FTD.

When interpreting our findings, it is important to keep in mind that CD68 staining marks the lysosomal compartment in these cells [35]. In this context, our results suggest that in vivo TSPO–PET signal is enriched in association with a microglial/macrophage population with elevated CD68 signals, rather than a tissue-wide expansion of microglial density. Single-cell Imaris data support this interpretation: we found that IBA1+ cells which were also CD68+ were both larger in volume and expressed more absolute TSPO than CD68- cells, though the IBA1–TSPO volume ratio remained unchanged. This is consistent with recent studies which compared TSPO expression in single-marker labelled microglia: Nutma et al. [30] showed that in human neurodegenerative tissue microglial TSPO was not up-regulated as a fraction of cell area when comparing cells labelled with IBA1, CD68 or HLA–DR. Our previous work on PSP reached the same conclusion, attributing the disease-related TSPO increase to microglial burden rather than per-cell up-regulation [41]. However, in both studies, TSPO was only analysed relative to the cell-marker area. Here, we report an increase of absolute TSPO in populations detected by IBA1 and CD68 in FTD suggesting an increased expression of TSPO in microglial cells with a phagocytic phenotype. Garland et al. [11] also found CD68+ microglia to be the dominant TSPO-expressing population in Alzheimer’s disease, in keeping with our observation that CD68+ microglia are preferentially associated with TSPO. Set against the greater CD68 burden in the pathological groups, the concordance between this *post-mortem* signal and in vivo TSPO–PET, and the persistence of elevated TSPO to the point of death, these findings position CD68+ microglia as the cells most closely associated with the in vivo TSPO signal within our experiments.

As an exploratory analysis in pathology-specific sub-cohorts, we tested the association between phagocytic–microglial burden (CD68) and tau or TDP-43 burden at post-mortem. Within the FTLD-tau sub-cohort, tau burden was modestly associated with CD68 burden across brain regions pre FDR correction. There was no association between pTDP-43 and CD68 in the FTLD–TDP sub-cohort. The CD68-tau coupling is partially concordant with prior in vivo TSPO-tau correspondences observed in multitracer PET studies in patients with primary tauopathies [2, 4, 25]. In these studies, TSPO–PET signal was regionally correlated with tau PET binding; however, the TSPO signals often extended beyond the tau epicentres. The absence of a CD68–pTDP-43 coupling does not discard a temporally contingent in vivo TSPO–TDP relationship, as TDP-43–PET tracers remain in development [40]. Of note, these exploratory analyses in pathology-specific sub-cohorts need to be interpreted with caution given the small per-subgroup sample sizes.

This study has several limitations. Due to the rarity of post-mortem tissue availability in individuals with TSPO–PET scans, while the sample sizes are larger than other PET-to-post-mortem studies this can still limit the power to detect small effects, and limits the interpretation of null findings. We accounted for this in several ways, including the implementation of linear mixed-effects models of all regional data points, and the use of FDR correction for interpretational robustness. [^11^C]PK11195 is a first-generation TSPO tracer with a lower signal-to-noise ratio compared to second- and third-generation TSPO tracers [24]. However, these alternative tracers are confounded by common genetic polymorphisms that substantially affect binding, making them less suitable for studies of rare diseases, where gene-stratified recruitment would be especially challenging. Further studies could aim to replicate our approach with other TSPO tracers. The antibodies used carry their own constraints as IBA1 immunoreactivity purportedly degrades in disease-affected tissue [15], and CD68 identifies the lysosome-enriched microglial compartment without completely resolving specific functional sub-states [33], or inferences regarding lysosomal functional integrity [35]. A further limitation is that cerebral post-mortem tissue was sampled from one hemisphere only, whereas cortical BPND estimates were bilateral. In FTD, where pathology, atrophy, and inflammation may be lateralized, unilateral tissue sampling may not recapitulate the full pathology. Expanding PET-to-post-mortem studies with novel TSPO tracers and deeper microglial phenotyping will be necessary to resolve the specific cellular programmes captured by elevated TSPO–PET signals in neurodegeneration.

In conclusion, we have shown that the elevated [^11^C]PK11195 signal reflects increased CD68-immunoreactive and phagocytic–microglial signal in both FTLD-tau and FTLD–TDP forms of frontotemporal dementia. TSPO+ microglial cells contribute to in vivo regional TSPO–PET signal, over and above other cell types. We suggest that TSPO–PET can be interpreted as a microglial signal in FTD and related disorders, empowering experimental medicine studies and clinical trials of disease-modifying therapies.

## Supplementary Information

Below is the link to the electronic supplementary material.

Supplementary file1 (DOCX 1557 kb)

## Data Availability

The data supporting the findings of this study are available from the corresponding author on reasonable request. Anonymised regional TSPO PET binding values and quantitative neuropathology measurements can be shared with qualified investigators, subject to the governance requirements of the Cambridge Brain Bank and the relevant Research Ethics Committee approvals. Post-mortem human brain tissue is available through the Cambridge Brain Bank in accordance with its tissue access policy.

## Code availability

The R analysis code used to generate the results reported here is available from the corresponding author on reasonable request.
