# Supplementary material for: TSPO–PET binding in vivo reflects increased phagocytic microglia at post mortem in people with frontotemporal dementia
Source: Acta Neuropathol. 2026 Oct 22;152(1):59. doi: 10.1007/s00401-026-03103-5 (PMC13690867; doi:10.1007/s00401-026-03103-5)
Supplement: Supplementary file 1 [file 401_2026_3103_MOESM1_ESM.docx]

**Electronic Supplementary Material — Online Resource 1**

Article: TSPO PET binding in vivo reflects increased phagocytic microglia at post mortem in people with frontotemporal dementia

Davi S. Vontobel^1,2^, Kei Onn Lai^1,2^, Mehtap Bacioglu^1^, George Nolan^1^, Daniel C Maddison^1,2^, Amelie Adamski**^1^**, Julia Goddard^1,2^, Noah L. Shapiro^1,2^, Harry Crook^1,2^, Tim Fryer^1^, Young Hong^1^, Sasvi Wijesinghe**^1^**, Franklin Aigbirhio^1^, Edward Avezov^1,2^, Kieren S. J.  Allinson^1,4^, Annelies Quaegebeur^1,4,5^, John T O’Brien^3^, James B Rowe^1,4^$\dagger$, Maria Grazia Spillantini^1^$\dagger$, Maura Malpetti^1,2,4^$\dagger$

Corresponding author: Prof. Maura Malpetti, mm2243@medschl.cam.ac.uk

# Online Resource 1 (Electronic Supplementary Material)

## A1. Imaris sub-cohort and pathology-class assignment

The full demographic, clinical and neuropathological cohort information is in **Table 1**. This appendix summarises the pathology-subgroup assignment used in the Tau / TDP analyses (section A2) and the Imaris sub-cohort (Fig. 5).

| ID | Group | Pathology subgroup | PET-PM Interval (Months) | In Imaris cohort |
| --- | --- | --- | --- | --- |
| Patient 1 | FTD | FTLD-tau | 91.0 | yes |
| Patient 2 | FTD | FTLD-tau | 124.0 | yes |
| Patient 3 | FTD | FTLD-TDP | 50.0 | yes |
| Patient 4 | FTD | FTLD-tau | 56.0 | yes |
| Patient 5 | FTD | FTLD-TDP | 100.0 | yes |
| Patient 6 | FTD | FTLD-TDP | 63.0 | yes |
| Patient 7 | FTD | FTLD-TDP | 54.0 | yes |
| Patient 8 | FTD | FTLD-TDP | 86.0 | yes |
| Patient 9 | FTD | FTLD-tau | 36.0 | yes |
| Patient 10 | FTD | FTLD-tau | 7.0 | yes |
| Control 1 | Control | - | - | yes |
| Control 2 | Control | - | - | yes |
| Control 3 | Control | - | - | no |
| Control 4 | Control | - | - | yes |
| Control 5 | Control | - | - | yes |

## A2. Aggregate (Tau / pTDP-43) ~ CD68

In an exploratory analysis, within each FTLD class, protein-aggregate burden tracked microglial load weakly. Each subtype was fitted separately by LMEM (response = aggregate %DAB, fixed predictor = CD68 %; *n* = 5 per group), with random effects chosen by the Matuschek ladder. Only Tau demonstrated a weak correlation with CD68 before FDR correction.

• (a) FTLD-tau — tau %DAB rose modestly with CD68 (LMEM [RI]: β = +0.0552, SE = 0.0265, *t* = 2.09, *P* = 0.039, *P*_FDR = 0.079; R²m = 0.032).

• (b) FTLD-TDP — pTDP-43 %DAB was unrelated to CD68 (LMEM [RI]: β = -0.1074, SE = 0.0905, *t* = -1.19, *P* = 0.241, *P*_FDR = 0.241; R²m = 0.027).

**Fig. S1 Protein-aggregate burden against CD68 burden across all regions in FTLD-tau patients (a) and FTLD-TDP patients (b), separately.** **(a)** Tau %DAB ~ CD68 in FTLD-tau (n = 5); β = +0.0552, SE = 0.0265, t = 2.09, *P* = 0.039, *P*_FDR = 0.079. **(b)** pTDP-43 %DAB ~ CD68 in FTLD-TDP (n = 5); β = -0.1074, SE = 0.0905, t = -1.19, *P* = 0.241, *P*_FDR = 0.241. Per-subset LMEM with Matuschek-selected RE (both subsets selected RI).

## A3. Tissue TSPO area fraction × BPND × Segmentation - Figure S2

Splitting tissue into grey and white matter did not improve on the corresponding whole-tissue model, so the TSPO area-fraction–BPND relationship holds across both compartments rather than being driven by one. Segmentation main effect (LRT 1-vs-2); not preferred over the BPND-only baseline (χ² = 0.224, df = 1, *P* = 0.636). BPND × Segmentation interaction (LRT 2-vs-3); not preferred over the additive model (χ² = 0.337, df = 1, *P* = 0.562). GM slope (REML refit of rung 3); β = +0.0560, SE = 0.0404, *t* = 1.39, *P* = 0.168, *P*_FDR = 0.233 (Benjamini–Hochberg within the 2-slope family). WM slope — β = +0.0303, SE = 0.0252, *t* = 1.20, *P* = 0.233, *P*_FDR = 0.233. GM − WM contrast — slopes look distinct but do not differ statistically (+0.0257, SE = 0.0482, *t* = 0.53, *P* = 0.595).

See also Fig. S3 for the per-marker (CD68 / IBA1 / GFAP / CD31) segmentation cascade on the marker-TSPO co-localised area.

**Fig. S2 Tissue TSPO area fraction × BPND, decomposed by tissue segmentation.** Tissue TSPO area fraction modelled by LMEM (*Post mortem TSPO ~ TSPO PET * Segmentation + SlideSet + PET_PM_Interval + (1|id)*) within the 10 patients with FTD. Per-Segmentation BPND slopes: GM β = +0.0560 (SE = 0.0404, t = 1.39, P = 0.168); WM β = +0.0303 (SE = 0.0252, t = 1.20, P = 0.233). The grey- versus white-matter slope contrast is +0.0257 (SE = 0.0482, t = 0.53, P = 0.595), i.e. the BPND–area-fraction association does not differ between compartments. Supporting nested-model tests: Segmentation main effect χ² = 0.224, df = 1, P = 0.636; BPND × Segmentation χ² = 0.337, df = 1, P = 0.562.

## A4. Coloc × Segmentation - GM-driven decomposition - Figure S3

A grey-matter / white-matter decomposition (Supplementary Figure S3) shows the CD68 positive coupling concentrated in grey matter (GM β = +0.013, P = 0.052; WM β = −0.009, P = 0.027; TSPO PET signal × Segmentation interaction LRT P = 0.007; GM–WM slope contrast P = 0.006, P_FDR = 0.012), with the same compartment-specific structure for IBA1 (GM β = +0.048, P = 0.006; WM β = −0.016, P = 0.122; LRT P = 0.001; contrast P = 0.002, P_FDR = 0.008). GFAP and CD31 show no significant compartmental difference (LRT P = 0.310 and 0.88; contrast P = 0.281 and 0.951; P_FDR = 0.375 and 0.951).

| Marker | LRT additive vs bpnd_only | LRT interaction vs additive | GM slope (BPND) | WM slope (BPND) | GM - WM contrast |
| --- | --- | --- | --- | --- | --- |
| **CD68** | *P* = 0.082 | ***P* = 0.007** | β = +0.0131, *P* = 0.052 | β = -0.0093, *P* = 0.027 | β = +0.0224, *P* = 0.006 |
| **IBA1** | *P* = 0.215 | ***P* = 0.001** | β = +0.0483, *P* = 0.006 | β = -0.0164, *P* = 0.122 | β = +0.0648, *P* = 0.002 |
| GFAP | *P* = 0.522 | *P* = 0.310 | β = +0.0051, *P* = 0.785 | β = -0.0192, *P* = 0.106 | β = +0.0243, *P* = 0.281 |
| CD31 | ***P* = 0.019** | *P* = 0.880 | β = +0.0077, *P* = 0.604 | β = +0.0088, *P* = 0.346 | β = -0.0011, *P* = 0.951 |

**Fig. S3** Marker–TSPO co-localised area × BPND, decomposed by tissue segmentation. Marker–TSPO co-localised area modelled by LMEM with regional TSPO PET binding (BPND) as the fixed-effect predictor, within all 10 patients with FTD, decomposed by grey and white matter (*Coloc_by_Total ~ BPND * Segmentation + PET_PM_Interval + (1|id)*). (a) CD68. (b) IBA1. (c) GFAP. (d) CD31. Per-segmentation points and linear-fit slope ribbons are coloured GM = grey (filled circles), WM = black (filled triangles). Per-compartment BPND slopes (β) with their p-values are estimated marginal trends (emtrends). pGM–WM is the model-based test of the grey- versus white-matter BPND-slope difference (the BPND × Segmentation contrast); pGM–WM,FDR is its Benjamini–Hochberg-adjusted value across the four markers (family m = 4). The CD68 and IBA1 slopes differ significantly between compartments (pGM–WM,FDR = 0.012 and 0.008), with positive GM and negative WM coupling; GFAP and CD31 show no compartmental difference (pGM–WM,FDR = 0.375 and 0.951).

## A5. Cell marker tissue-fraction companion to the co-localisation analysis

In the same slides as the co-localisation analysis, CD68 and IBA1 co-localised marker area diverge in direction across regions while GFAP and CD31 did not, reproducing the same pattern. Random intercept (Matuschek-selected; Marker Area ~ TSPO PET + PET_PM_Interval + (1|id)) for all four markers, with BH correction.

• (a) CD68 — β_BPND = +0.0465, SE = 0.0089, *t* = 5.25, *P* < 0.001, *P*_FDR < 0.001.

• (b) IBA1 — β_BPND = -0.0720, SE = 0.0156, *t* = -4.62, *P* < 0.001, *P*_FDR < 0.001.

• (c) GFAP — β_BPND = -0.1394, SE = 0.0463, *t* = -3.01, *P* = 0.004, *P*_FDR = 0.005.

• (d) CD31 — β_BPND = -0.0209, SE = 0.0076, *t* = -2.74, *P* = 0.008, *P*_FDR = 0.008.

**Fig. S4 Cell marker tissue fraction × TSPO PET, four-marker companion.** Marker area expressed as a fraction of total tissue modelled by LMEM, fitted per marker. LMEMs Matuschek-selected; Marker Area ~ TSPO PET + PET_PM_Interval + (1|id) **(a)** CD68: β_BPND = +0.0465, SE = 0.0089, t = 5.25, *P* < 0.001, *P*_FDR < 0.001. **(b)** IBA1: β_BPND = -0.0720, SE = 0.0156, t = -4.62, *P* < 0.001, *P*_FDR < 0.001. **(c)** GFAP: β_BPND = -0.1394, SE = 0.0463, t = -3.01, *P* = 0.004, *P*_FDR = 0.005. **(d)** CD31: β_BPND = -0.0209, SE = 0.0076, t = -2.74, *P* = 0.008, *P*_FDR = 0.008. RE = RI selected by Matuschek for all four markers.

**Fig. S5 Subject-level single-cell Imaris metrics do not differ between groups.** Colour palette: Control = grey, FTLD-tau = orange, FTLD-TDP = blue; box fill salmon = FTD, light grey = Control; points coloured by pathology subgroup. Per-subject values compared between FTD and Control by Wilcoxon rank-sum test (grey and white matter pooled), with Hodges–Lehmann shift and 95% CI; BH-FDR across the four-test family (m = 4). (a) IBA1+ cell density per subject; W = 28, P = 0.288, P_FDR = 0.454, HL = +25.6 [−22, +94]. (b) Intracellular TSPO volume fraction in IBA1+ microglia; W = 13, P = 0.374, P_FDR = 0.454, HL = −0.019 [−0.039, +0.021]. (c) Intracellular CD68 volume fraction in IBA1+ microglia; W = 32, P = 0.106, P_FDR = 0.424, HL = +0.0019 [−0.0006, +0.0065]. (d) Intracellular TSPO volume fraction within CD68+ cells; W = 14, P = 0.454, P_FDR = 0.454, HL = −0.010 [−0.049, +0.022]. (e) Proportion of IBA1+ microglia that were CD68+ per subject (grey and white matter pooled); W = 33, P = 0.076, HL = +0.19 [−0.07, +0.38]. Panel (e) was added at revision and was tested as a single-test family of its own, so its P value is not part of the four-test Benjamini–Hochberg family applied to panels (a)–(d).

## A6. Antibody tables (primary and secondary)

**Table S5. Primary antibodies used for immunohistochemistry (IHC) and immunofluorescence (IF).**

| Protocol | Target | Species | Dilution | Antigen retrieval buffer | Supplier (cat. no.) | Target description |
| --- | --- | --- | --- | --- | --- | --- |
| IHC | CD68 | Mouse | 1:50 | Sodium citrate (pH 6.2) | Dako (M0876) | Microglial lysosomes |
| IHC | AT8 | Mouse | 1:500 | Sodium citrate (pH 6.2) | Invitrogen (MN1020) | Phospho-tau (Ser202, Thr205) |
| IHC | TDP-43 | Mouse | 1:1000 | Sodium citrate (pH 6.2) | CosmoBio (CAC-TIP-PDT-M01A) | Phosphorylated TDP-43 (Ser409/410) |
| IF | TSPO | Rabbit | 1:500 | Tris-EDTA (pH 9.0) | Abcam (ab109497, clone EPR5384) | Mitochondrial protein |
| IF | GFAP | Goat | 1:200 | Tris-EDTA (pH 9.0) | Antibodies.com (A83720) | Astrocytic structural protein |
| IF | AIF1/IBA1 | Goat | 1:250 | Tris-EDTA (pH 9.0) | Antibodies.com (A82670) | Microglial membrane-ruffling protein |
| IF | CD68 | Mouse | 1:250 | Tris-EDTA (pH 9.0) | Dako (M0876) | Microglial lysosomes |
| IF | CD31 | Mouse | 1:500 | Tris-EDTA (pH 9.0) | Abcam (ab9498) | Endothelial lumen |

**Table S6. Secondary antibodies.**

| Protocol | Target species | Host | Dilution | Fluorophore | Supplier (cat. no.) |
| --- | --- | --- | --- | --- | --- |
| IHC | Mouse | Donkey | 1:500 | n/a (HRP) | ThermoFisher (A24506) |
| IF | Rabbit | Donkey | 1:500 | AF568 | ThermoFisher (A10042) |
| IF | Goat | Donkey | 1:500 | AF488 | ThermoFisher (A-11055) |
| IF | Mouse | Donkey | 1:500 | AF647 | ThermoFisher (A-31571) |

## A7. Compartment-restricted CD68 group models

The segmentation-aware companion model reported in the main text partitions the group contrast across every region-by-compartment cell, which is conservative for the grey-matter comparison. As an additional analysis at revision, CD68 %DAB area was therefore modelled separately within each compartment, across the seven regions for which the segmentation provides a grey/white split (BA1/2/3, BA6, BA7, BA17/18, BA21/22, BA46 and cerebellar cortex). Each model was CD68 ~ Group + Region + (1|id), fitted by REML with Type III F-tests and Kenward–Roger denominator degrees of freedom, on N = 98 observations from 15 subjects (5 controls, 10 patients with FTD) per compartment. Images within a subject–region–segmentation cell were averaged before modelling, so the unit of observation is the subject–region cell.

In grey matter the group effect was significant (F(1, 13.5) = 8.48, P_raw = P_BH = 0.012; the group term is the sole member of its correction family), with β (FTD − Control) = +1.07 %DAB, SE = 0.37, 95% CI [+0.28, +1.87]; control mean 1.58 %DAB versus 2.64 %DAB in FTD. In white matter the group effect was larger in absolute terms (F(1, 13.5) = 11.38, P_raw = P_BH = 0.005), with β = +2.50 %DAB, SE = 0.74, 95% CI [+0.91, +4.10]; control mean 4.35 %DAB versus 6.86 %DAB in FTD. Adding a Group × Region interaction improved neither model (grey matter LRT χ²(6) = 7.72, P = 0.260; white matter χ²(6) = 6.44, P = 0.376), and the interaction term was not significant in either compartment (grey matter F(6, 72.2) = 1.15, P = 0.342; white matter F(6, 72.1) = 0.96, P = 0.462). The elevation is therefore better described as broadly distributed across the sampled regions than as regionally selective.

Per-region FTD − Control contrasts from the interaction model are given below for each compartment, with Benjamini–Hochberg adjustment applied across the family of seven regions within that compartment. Both uncorrected and adjusted P-values are reported.

| **Compartment** | **Region** | **β (FTD − Control), %DAB** | **SE** | **df** | **95% CI** | **P_raw** | **P_BH** |
| --- | --- | --- | --- | --- | --- | --- | --- |
| Grey matter | BA46 | +1.622 | 0.570 | 55.6 | +0.479, +2.765 | 0.0062 | 0.0218 |
| Grey matter | BA6 | +1.622 | 0.570 | 55.6 | +0.480, +2.763 | 0.0062 | 0.0218 |
| Grey matter | BA21/22 | +1.246 | 0.571 | 55.5 | +0.101, +2.390 | 0.0335 | 0.0781 |
| Grey matter | BA7 | +1.151 | 0.571 | 55.5 | +0.006, +2.295 | 0.0488 | 0.0854 |
| Grey matter | BA1/2/3 | +1.094 | 0.621 | 63.9 | −0.148, +2.335 | 0.0831 | 0.1164 |
| Grey matter | BA17/18 | +0.550 | 0.538 | 49.2 | −0.530, +1.630 | 0.3114 | 0.3633 |
| Grey matter | Cerebellar cortex | +0.333 | 0.570 | 55.6 | −0.810, +1.476 | 0.5619 | 0.5619 |
| White matter | BA6 | +3.211 | 1.139 | 54.2 | +0.927, +5.494 | 0.0067 | 0.0244 |
| White matter | BA7 | +3.203 | 1.142 | 54.0 | +0.913, +5.492 | 0.0070 | 0.0244 |
| White matter | BA46 | +3.014 | 1.140 | 54.1 | +0.728, +5.300 | 0.0107 | 0.0250 |
| White matter | BA1/2/3 | +2.795 | 1.241 | 62.6 | +0.315, +5.275 | 0.0278 | 0.0422 |
| White matter | BA21/22 | +2.543 | 1.142 | 54.0 | +0.254, +4.832 | 0.0301 | 0.0422 |
| White matter | Cerebellar cortex | +2.394 | 1.140 | 54.1 | +0.108, +4.680 | 0.0405 | 0.0472 |
| White matter | BA17/18 | +0.755 | 1.076 | 47.8 | −1.408, +2.918 | 0.4860 | 0.4860 |

Regions are ordered by uncorrected P within each compartment. In white matter six of the seven regions remain below 0.05 after Benjamini–Hochberg adjustment; in grey matter BA46 and BA6 do, with BA21/22 and BA7 falling between 0.05 and 0.09.

## A8. Model specifications

Table S7. Linear mixed-effects model specifications for all analyses reported in the Results, in order of appearance. Model formulas are given in lme4 notation; RE = random-effects structure (RI = random intercept, (1|id)); selection by the Matuschek ladder where indicated. Observation counts for models M5–M8 vary by slide set and by marker and are therefore not tabulated.

| **#** | **Results section** | **Outcome** | **Model formula** | **RE / selection** | **N obs** | **N subjects** |
| --- | --- | --- | --- | --- | --- | --- |
| M1 | CD68 burden group contrast | CD68 %DAB area | CD68 ~ Group × Region + (1\|id) | RI (a priori) | 323 | 15 |
| M2 | CD68 segmentation companion | CD68 %DAB area | CD68 ~ Group × Region × Segmentation + (1\|id) | RI (a priori) | 323 | 15 |
| M3 | BPND–CD68 coupling | CD68 %DAB area | CD68 ~ BPND + PET_PM_Interval + (1\|id) | Matuschek RI | 247 | 10 |
| M4 | Pathology-subgroup slopes | CD68 %DAB area | CD68 ~ PathClass × BPND + PET_PM_Interval + (1\|id); LRT against the reduced model CD68 ~ PathClass + BPND + PET_PM_Interval + (1\|id) | Matuschek RI | 247 | 10 |
| M5 | Tissue TSPO group contrast (per slide set) | TSPO area fraction | Norm_AreaB ~ Group + Region + (1\|id) | RI (a priori) |  | 15 |
| M6 | Tissue TSPO–BPND joint coupling | TSPO area fraction | Norm_AreaB ~ BPND × SlideSet + PET_PM_Interval + (1\|id) | Matuschek RI |  | 10 |
| M7 | Marker–TSPO co-localisation group contrast (four markers) | Coloc / Total area | Coloc ~ Group + Region + (1\|id) | RI (a priori) |  | 15 |
| M8 | Marker–TSPO coloc–BPND coupling (four markers) | Coloc / Total area | Coloc ~ BPND + PET_PM_Interval + RE | Matuschek: CD68, IBA1, CD31 RI; GFAP (1 + BPND\|id) |  | 10 |
